# Bioactive Compounds from Natural Sources: Discovery, Evaluation, and Applications

**DOI:** 10.3390/molecules29133209

**Published:** 2024-07-05

**Authors:** Tao Liu, Xuexiang Chen

**Affiliations:** 1 Guangdong Provincial Key Laboratory of Pharmaceutical Bioactive Substances, School of Basic Medical Sciences, Guangdong Pharmaceutical University, Guangzhou 510006, China; 2 Department of Nutrition and Food Hygiene, School of Public Health, Guangzhou Medical University, Guangzhou 511436, China

Natural products are of paramount importance due to their extensive range of biological activities, making them indispensable in drug discovery and development. Their unique and complex chemical structures often serve as templates for designing and synthesizing new therapeutic agents. Notable examples include paclitaxel, artemisinin, morphine, and quinine, which are among the most active and representative components derived from natural sources. These compounds have immense value in treating major diseases: paclitaxel is primarily used in cancer treatment; artemisinin is crucial for malaria therapy; morphine is a potent analgesic widely used for pain management; and quinine is essential for treating malaria. Beyond pharmaceuticals, natural products play a crucial role in agriculture by providing bioactive compounds for crop protection and enhancement. In the food industry, they contribute to food safety and preservation, while in cosmetics, they offer natural ingredients for skincare and personal care products. Furthermore, the study of natural products aids in elucidating ecological interactions and biological processes, thereby fostering advancements in biotechnology and sustainable practices. The multifaceted applications and contributions of natural products highlight their critical significance in scientific research and technological innovation [1,2].

However, the journey from natural source to application is fraught with challenges. Bioactive compounds are often found within complex matrices, complicating their discovery, purification, and characterization. The intricate nature of these matrices necessitates advanced purification techniques and sophisticated analytical tools. Techniques such as liquid chromatography, pressurized liquid extraction, microwave-assisted extraction, and supercritical fluid extraction provide efficient and precise methods for compound separation. Simultaneously, advanced spectroscopic technologies, such as nuclear magnetic resonance (NMR), infrared (IR) spectroscopy, and mass spectrometry (MS), have enabled more accurate and comprehensive structure elucidation. These technologies not only improve research efficiency but also contribute significantly to the expansion of the compound library, laying a solid foundation for subsequent bioactivity studies.

In terms of bioactivity evaluation, natural products have shown a wide range of potential therapeutic effects. For instance, certain plant extracts can exert anti-inflammatory effects by inhibiting the release of inflammatory mediators and blocking inflammatory signaling pathways. Marine-derived compounds have demonstrated strong antibacterial and antiviral activities, making them promising candidates for anti-infective treatments. Moreover, natural products show great potential in neuroprotection, cardiovascular disease prevention, and metabolic disease treatment. For example, plant polyphenols, through their antioxidant and anti-inflammatory properties, can effectively protect neuronal cells from damage and prevent neurodegenerative diseases. Some natural compounds can regulate lipid and glucose levels, aiding in the prevention and treatment of cardiovascular and metabolic disorders [3,4,5].

With the development of computational biology and systems biology, natural prod-uct research has increasingly moved towards multidisciplinary integration. By combining molecular docking, molecular dynamics simulations, and other computational tech-niques, researchers can predict and optimize the bioactivity of natural compounds, en-hancing screening efficiency. Systems biology approaches allow researchers to under-stand the mechanisms of natural products at a holistic level, revealing their multi-target action modes in complex biological systems. Furthermore, the integration of artificial in-telligence (AI) into natural product research has injected new momentum into the field. Machine learning and deep learning algorithms enable researchers to quickly identify po-tential bioactive compounds from large datasets and automate structure prediction and bioactivity evaluation, significantly improving research efficiency and accuracy [6,7,8].

This Special Issue, “Bioactive Compounds from Natural Sources: Discovery, Evaluation, and Applications”, has successfully collected important contributions in the field of bioactive compounds from plants, microbes, and animals. The collection highlights recent breakthroughs and innovative approaches in the discovery, isolation, characterization, and application of these compounds. Contributions include studies on new bioactive compounds with potential therapeutic applications, advancements in isolation and analytical techniques, genome mining, and engineering approaches, as well as case studies demonstrating the application of these compounds in addressing major health challenges. The identification of novel polysaccharides with immunomodulatory activity from plant sources and the development of efficient synthetic protocols for bioactive compounds unique to certain herbs exemplify the breadth of research and innovation in this field.

Xia et al. investigated the chemical constituents of Hypericum seniawinii Maxim., isolating two new benzophenone glycosides (hypersen A and B) and four known compounds, including chromones and biflavonoids. The structures of these compounds were elucidated through comprehensive spectroscopic analysis, with their absolute configurations determined using electronic circular dichroism (ECD) calculations. Evaluating the biological activities of the isolated compounds revealed significant neuroprotective effects against corticosterone-induced PC12 cell injury. Additionally, compound 6 exhibited notable anti-inflammatory activity by significantly inhibiting nitric oxide production in lipopolysaccharide-induced RAW 264.7 cells, with an IC50 value of 11.48 ± 1.23 µM. This study enriches the chemical diversity of H. seniawinii, highlighting its potential for developing anti-inflammatory and neuroprotective agents.

Wang et al. explored the antitumor properties of prodigiosin (PG), a metabolite from Serratia marcescens found in cockroaches, against hepatocellular carcinoma (HCC) cells. The study demonstrated that PG inhibits the proliferation of HepG2 and BEL-7402 cells in a dose-dependent manner. PG induces apoptosis through endoplasmic reticulum stress (ERS), evidenced by increased intracellular Ca^2+^ and upregulation of ERS-related proteins (PERK, IRE1α, Bip, and CHOP) and apoptotic proteins (caspase3, caspase9, and Bax), while downregulating anti-apoptotic Bcl-2. In vivo experiments confirmed that PG reduces tumor growth without significant toxicity to major organs, highlighting its potential as a therapeutic agent for HCC.

Hou et al. identified a novel pectic polysaccharide (HPP-1) from immature honey pomelo fruit (Citrus grandis) that exhibits significant immunomodulatory activity. HPP-1 has a molecular weight of 59,024 Da and is composed of rhamnose, arabinose, fucose, mannose, and galactose. Structural analysis revealed the presence of both α- and β-glycosidic linkages. HPP-1 enhances the production of nitric oxide (NO), TNF-α, and IL-6 in macrophage RAW 264.7 cells. These immunomodulatory effects are mediated through the activation of NF-κB and MAPK signaling pathways, suggesting HPP-1 as a potential candidate for functional foods and immunological treatments.

Liu et al. developed an efficient synthesis protocol for five macamides, bioactive compounds unique to maca, achieving over 95% purity. The synthesized macamides were tested for their anti-fatigue activity using a forced swimming test (FST) in mice. The results showed that N-benzyl-hexadecanamide (NBH), the most abundant macamide, significantly increased endurance capacity by raising liver glycogen levels and reducing blood urea nitrogen, lactate dehydrogenase, blood ammonia, and blood lactic acid levels. The study demonstrated the potential of macamides to alleviate physical fatigue and improve exercise performance.

Ma et al. conducted a phytochemical investigation of *Camellia ptilosperma* leaves, isolating ten novel compounds, including six triterpenes and four pheophorbides. The triterpenes demonstrated significant cytotoxic activity against several cancer cell lines, with compound **2** showing potent activity against HepG2 cells (IC50 = 2.57 µM). Additionally, compounds **4** and **5** exhibited cytotoxicity against MDA-MB-231 cells. The pheophorbides displayed notable photocytotoxic and photodynamic antibacterial activities. Specifically, compound **7** showed exceptional photocytotoxicity against HeLa, MCF-7, and A549 cells, and compound **10** demonstrated significant photodynamic cytotoxicity against BEL-7402 and HepG2 cells. The antibacterial activity was also notable, with compounds **8** and **10** showing effectiveness against *E. coli*. These findings suggest that *C. ptilosperma* has potential medicinal applications due to its bioactive compounds.

Lv et al. described an efficient iodine-mediated one-pot synthesis method for pyrrolo/indolo [1,2-a]quinoxalines and quinazolin-4-ones using epoxides as alkyl precursors under metal-free conditions. This method is applicable to both 1-(2-aminophenyl)-pyrrole and 2-aminobenzamide, producing 33 desired products with moderate-to-good yields. The practicality of the method is demonstrated through its scalability and potential for further modification into pharmaceutically active compounds. Leveraging the Meinwald rearrangement of epoxides, this approach allows the formation of valuable heterocycles that exhibit significant biological activities, such as antimalarial, anticancer, and antibacterial properties, making it a versatile and practical advancement in green synthesis.

Liu et al. developed a highly efficient one-pot synthetic protocol for creating benzofuran and benzo [b]thiophen derivatives under transition-metal-free conditions at room temperature. This method proved advantageous due to its mild reaction conditions, simple procedure, and broad substrate scope, achieving good-to-excellent yields of regioselective five-membered heterocycles. The process was particularly noted for its green and clean synthetic methodology, making it suitable for the synthesis of biologically and medicinally relevant compounds. The authors highlighted the synthesis of various derivatives, emphasizing the method’s potential in pharmaceutical applications.

Liu et al. identified three natural compounds—Poliumoside, Soyasaponin Bb, and Saikosaponin B2—as potential N-glycanase 1 (NGLY1) inhibitors through a structure-based virtual screening of 2960 natural compounds. These compounds share a core disaccharide structure of glucose and rhamnose, binding to key active sites Lys238 and Trp244 on NGLY1, which are crucial for the deglycosylation of misfolded glycoproteins in the ERAD pathway. The study highlights the potential of these inhibitors in developing antiviral and anticancer therapies. Poliumoside, Soyasaponin Bb, and Saikosaponin B2 were isolated from traditional Chinese medicinal herbs known for their heat-clearing and detoxifying properties. The core disaccharide motif found in these compounds serves as a promising foundation for future drug development, leveraging the structural insights for designing novel NGLY1 inhibitors with improved efficacy and safety profiles, particularly in the context of viral infections and cancer.

Huang et al. conducted a study to identify anti-hyperuricemia compounds from Cortex Fraxini using ABCG2 and bioaffinity ultrafiltration mass spectrometry (BA-UF-MS). The research focused on the ATP-binding cassette transporter ABCG2, which plays a crucial role in urate excretion and is a potential target for treating hyperuricemia. The study successfully identified fraxin as an active compound that binds to ABCG2. In vitro and in vivo experiments confirmed that fraxin activates ABCG2, thereby lowering serum uric acid levels in rats. This research highlights the efficacy of BA-UF-MS in rapidly screening and identifying active compounds in traditional Chinese medicine (TCM) and suggests fraxin as a promising candidate for hyperuricemia treatment.

Fan et al. isolated and characterized a polysaccharide, CPTM-P1, from Taxus media. The study detailed the extraction and purification process, revealing that CPTM-P1 is an acidic heteropolysaccharide with a molecular weight of 968.7 kDa. Compositional analysis identified ten monosaccharides, with galactose being the most abundant. Structural analysis using FT-IR and NMR confirmed the presence of uronic acids and pyranose rings, indicating a complex structure. Functionally, CPTM-P1 exhibited significant immunoenhancing properties, including the stimulation of nitric oxide and cytokine (TNF-α, IL-1β, and IL-6) production in RAW264.7 macrophage cells, suggesting potential applications in immunotherapy and functional foods.

In conclusion, research on bioactive compounds from natural sources holds significant scientific importance and offers extensive application prospects in exploring chemical diversity, evaluating biological activity, and achieving practical applications. Through continuous technological innovation and multidisciplinary integration, we are poised to discover more natural compounds with therapeutic potential, thereby advancing modern medicine and health science.

## References

[1] Rehman M.U., Abdullah, Khan F., Niaz K., Sanches Silva A., Nabavi S.F., Saeedi M., Nabavi S.M. (2020). Chapter 1—Introduction to natural products analysis. Recent Advances in Natural Products Analysis.

[2] Patra J.K., Das G., Lee S., Kang S., Shin H. (2018). Selected commercial plants: A review of extraction and isolation of bioactive compounds and their pharmacological market value. Trends Food Sci. Technol..

[3] Pereira L., Cotas J. (2023). Therapeutic Potential of Polyphenols and Other Micronutrients of Marine Origin. Mar. Drugs.

[4] Zhang Y., Cai P., Cheng G., Zhang Y. (2022). A Brief Review of Phenolic Compounds Identified from Plants: Their Extraction, Analysis, and Biological Activity. Nat. Prod. Commun..

[5] Ebrahimi B., Baroutian S., Li J., Zhang B., Ying T., Lu J. (2023). Combination of marine bioactive compounds and extracts for the prevention and treatment of chronic diseases. Front. Nutr..

[6] Gupta R., Srivastava D., Sahu M., Tiwari S., Ambasta R.K., Kumar P. (2021). Artificial intelligence to deep learning: Machine intelligence approach for drug discovery. Mol. Divers..

[7] Santana K., do Nascimento L.D., Lima e Lima A., Damasceno V., Nahum C., Braga R.C., Lameira J. (2021). Applications of Virtual Screening in Bioprospecting: Facts, Shifts, and Perspectives to Explore the Chemo-Structural Diversity of Natural Products. Front. Chem..

[8] Moshawih S., Goh H.P., Kifli N., Idris A.C., Yassin H., Kotra V., Goh K.W., Liew K.B., Ming L.C. (2022). Synergy between machine learning and natural products cheminformatics: Application to the lead discovery of anthraquinone derivatives. Chem. Biol. Drug Des..

